# Cryptic diversity and deep divergence in an upper Amazonian leaflitter frog, *Eleutherodactylus ockendeni*

**DOI:** 10.1186/1471-2148-7-247

**Published:** 2007-12-21

**Authors:** Kathryn R Elmer, José A Dávila, Stephen C Lougheed

**Affiliations:** 1 Department of Biology, Queen's University, Kingston, ON, K7L 3N6, Canada; 2 Instituto de Investigación en Recursos Cinegéticos, IREC (CSIC, UCLM, JCCM), Ronda de Toledo s/n, 13005 Ciudad Real, Spain; 3 Current address : Lehrstuhl für Zoologie und Evolutionsbiologie, Department of Biology, University of Konstanz, Universitätstraße 10, 78457 Konstanz, Germany

## Abstract

**Background:**

The forests of the upper Amazon basin harbour some of the world's highest anuran species richness, but to date we have only the sparsest understanding of the distribution of genetic diversity within and among species in this region. To quantify region-wide genealogical patterns and to test for the presence of deep intraspecific divergences that have been documented in some other neotropical anurans, we developed a molecular phylogeny of the wide-spread terrestrial leaflitter frog *Eleutherodactylus ockendeni *(Leptodactylidae) from 13 localities throughout its range in Ecuador using data from two mitochondrial genes (16S and cyt b; 1246 base pairs). We examined the relation between divergence of mtDNA and the nuclear genome, as sampled by five species-specific microsatellite loci, to evaluate indirectly whether lineages are reproductively isolated where they co-occur. Our extensive phylogeographic survey thus assesses the spatial distribution of *E. ockendeni *genetic diversity across eastern Ecuador.

**Results:**

We identified three distinct and well-supported clades within the Ecuadorean range of *E. ockendeni*: an uplands clade spanning north to south, a northeastern and central lowlands clade, and a central and southeastern clade, which is basal. Clades are separated by 12% to 15% net corrected p-distance for cytochrome *b*, with comparatively low sequence divergence within clades. Clades marginally overlap in some geographic areas (e.g., Napo River basin) but are reproductively isolated, evidenced by diagnostic differences in microsatellite PCR amplification profiles or DNA repeat number and coalescent analyses (in MDIV) best modelled without migration. Using Bayesian (BEAST) and net phylogenetic estimates, the Southeastern Clade diverged from the Upland/Lowland clades in the mid-Miocene or late Oligocene. Lowland and Upland clades speciated more recently, in the early or late Miocene.

**Conclusion:**

Our findings uncover previously unsuspected cryptic species diversity within the common leaflitter frog *E. ockendeni*, with at least three different species in Ecuador. While these clades are clearly geographically circumscribed, they do not coincide with any existing landscape barriers. Divergences are ancient, from the Miocene, before the most dramatic mountain building in the Ecuadorean Andes. Therefore, this diversity is not a product of Pleistocene refuges. Our research coupled with other studies suggests that species richness in the upper Amazon is drastically underestimated by current inventories based on morphospecies.

## Background

Species richness and genetic diversity within species are proposed to co-vary and understanding the details of this relationship is critical to unifying biodiversity theory [1]. Though the forests of the upper Amazon basin are a renowned hotspot for amphibian species richness [2], thus far there have been few explorations of the phylogeographic and population-level patterns in the region's amphibian taxa [3-9]. Consequently, we have only a preliminary knowledge of the distribution of genetic diversity from merely a handful of amphibian species in the megadiverse upper Amazon. With the aim of augmenting our understanding of spatial patterns of genetic diversity and details of evolutionary history in this famously species-rich area, we assessed the diversity in a widespread upper Amazonian frog species across the upper Amazon of Ecuador.

Historical and environmental characteristics of a region influence multiple levels of diversity, both in its origin and maintenance [1,10-12]. Numerous regional historical, topographical, and ecological factors of the Andes and Amazon have been suggested as influential in the diversification of species, for example: riverine barriers [13-16]; large uninterrupted area [10,17]; an Andean "species pump" promoted by complex, isolating montane topography and vegetation [18]; Pleistocene forest refuges [11,19-21]; complex biotic interactions [22,23]; ancient ridges and other palaeogeographic features [4,24]; ancientness [25]; and relative youth of the Andes [26]. For amphibians, complex topography may restrict vagility and produce marked population subdivision, and ultimately patterns of adaptive radiation or isolation patterns that are potentially implicated in high local rates of speciation [27]. There are many confounds in the upper Amazon that make exclusive testing of any one of these factors challenging because multiple causes at various historical timescales are surely involved in the origination of diversity [8,28]. Nonetheless, contributing temporal and geographical patterns of diversity in phylogeographic studies allows us to discern among some of the diversification hypotheses (e.g., ancientness versus youth, orogenesis events versus recent climatic change). Assessing these patterns is critical to disentangling the causes of within and among species diversification and the genesis of anuran biodiversity, especially as this region increasingly suffers deforestation.

Here we use mitochondrial and nuclear DNA to quantify the phylogeography and population structure of a terrestrial upper Amazonian leaflitter frog, *Eleutherodactylus ockendeni *(Leptodactylidae). We sampled from 13 localities across the species range in megadiverse eastern Ecuador to develop a thorough regional phylogeographic survey. Further, we used phylogenetic and multiple coalescent methods to estimate the depth of divergence among clades and suggest temporal context for the divergence revealed by our analyses.

## Results

### DNA sequences

We sequenced 105 individuals for cytochrome *b *(cyt *b*) and 45 individuals for 16S, plus two outgroup taxa (Additional file 1). Ingroup base frequencies are similar to those found in other frogs [29]: cyt *b*: A = 0.240, C = 0.313, G = 0.145, T = 0.301; 16S: A = 0.299, C = 0.251, G = 0.194, T = 0.255. Ingroup cyt *b *sequences collapsed into 21 unique haplotypes and 16S into 15 unique haplotypes. For the 16S-cytb data combined (32 individuals; 20 haplotypes and two outgroup taxa; 1248 bp), 227 included characters were parsimony informative.

### Phylogenetic analyses

**Figure 1:**
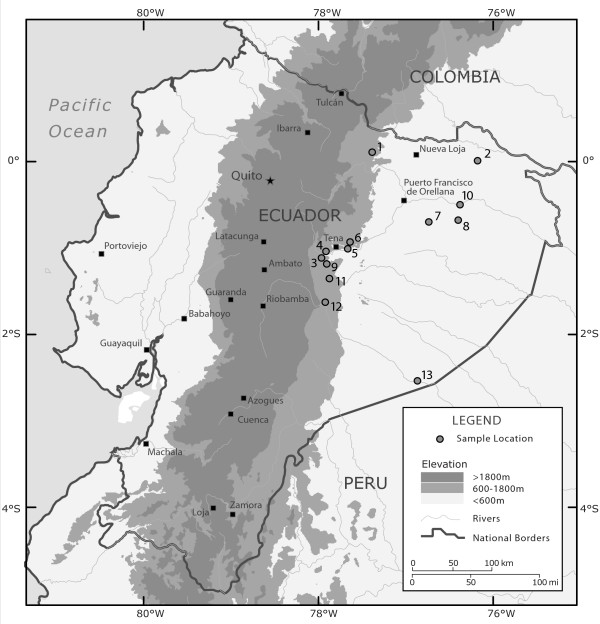
**Map of sample localities**. Map of sample localities across eastern Ecuador: Chonta Yacu (1), Reserva Cuyabeno (2), Serena (3), Cando (4), Jatun Sacha Biological Station (EBJS) and surrounding area (5), Puca Chicta (6), Auca 14 Road near Dayuma (7), Parque Nacional Yasuní (8), Llanganates mountains (9), La Selva Lodge (10), canton Santa Clara (11), Fundación Hola Vida (12), Kapawi Lodge (13).

**Figure 2:**
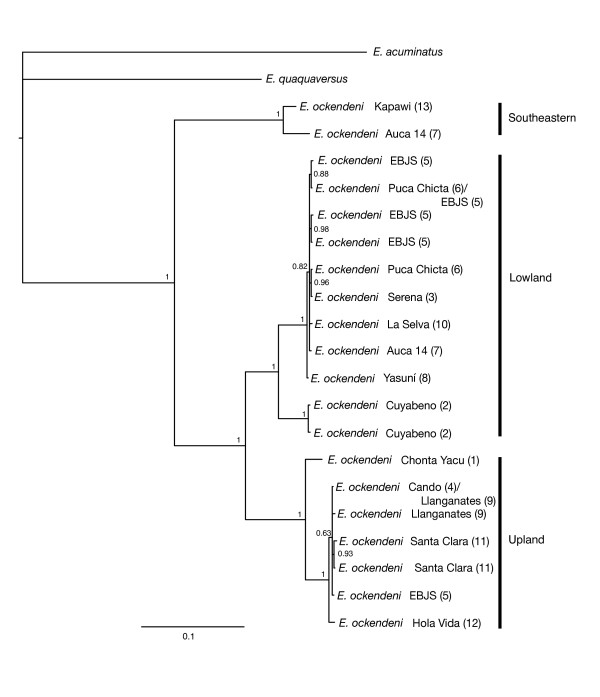
**Phylogenetic tree of Ecuadorean *E. ockendeni***. Bayesian phylogenetic tree of *E. ockendeni *samples and two outgroup taxa. Numbers in brackets correspond to localities in Figure 1. The topology and support were congruent with the MP tree. Posterior probabilities are labelled at nodes. The three major clades are noted.

We found no conflicting phylogenetic signal between the cyt *b *and 16S data (partition homogeneity test, *P *= 1.0), justifying the use of the combined data partition in maximum parsimony (MP) analyses. Both MP and Bayesian analyses of the 16S-cytb data produced well-supported trees identical in all major topological details and with three major clades: an Upland Clade (Figure 1 localities Hola Vida, Santa Clara, EBJS, Llanganates, Cando, and Chonta Yacu), a Lowland Clade (Yasuní, Puca Chicta, EBJS, Serena, Auca 14, La Selva, and Cuyabeno); and a Southeastern clade (Kapawi and Auca 14) (Figure 2). The topology of the 16S-cytb trees is congruent with trees resulting from separate analyses of the full 16S and cyt *b *data (results not shown).

### Population genetics

Corrected p-distances between *E. ockendeni *cyt *b *haplotypes were high, ranging up to 20% between some haplotypes (Additional file 2). Mean net divergence ± standard error [30] between the Southeastern Clade and the Upland Clade was 15.45% ± 1.83, between the Southeastern Clade and the Lowland Clade was 15.26% ± 1.90, and between Upland and Lowland Clades was 12.01% ± 1.72. Average sequence divergence was low within the Upland and Lowland clades, at 1.35 and 1.77% respectively, while the Southeastern Clade, which has fewer haplotypes and more geographically distant sampling, showed more intraclade diversity at 5.34%.

For the cyt *b *data, the identity of haplotypes varied among localities but seven of the 13 sites had only a single haplotype (Table 1). Nucleotide diversities within localities range from 0 to 0.105 ± 0.079. However, high nucleotide diversity within localities EBJS (5) and Auca 14 (7) is an artefact of finding sympatric but genetically distinct clades at those localities. When calculations of locality nucleotide diversity are separated by clade, the values range from 0 to 0.0030 ± 0.003 (Table 1). HKY+G was the best-fit model of nucleotide substitution for cyt *b *as chosen by hLRT.

**Table 1:** Population cyt *b *diversities

Locality	N	*n*	*h*	π	*s*	Shares with	Clade
Chonta Yacu (1)	5	1	0	0	0	none	Upland
Hola Vida (12)	5	1	0	0	0	none	Upland
Cando (4)	8	1	0	0	0	Llanganates, Sta Clara	Upland
Santa Clara (11)	5	4	0.9 ± 0.2	0.0028 ± 0.0022	4	Llanganates, Cando	Upland
Llanganates (9)	2	2	1.0 ± 0.5	0.0030 ± 0.0036	2	Cando, Santa Clara	Upland
EBJS (5)	55	6	0.6 ± 0.1	0.0171 ± 0.0088	69	Puca Chicta	Upland and Lowland
Upland	4	1	0	0	0	none	
Lowland	51	5	0.5 ± 0.7	0.0013 ± 0.0011	4	Puca Chicta	
Puca Chicta (6)	2	2	1.0 ± 0.5	0.0028 ± 0.0035	1	EBJS, Serena	Lowland
Serena (3)	5	1	0	0	0	Puca Chicta	Lowland
Cuyabeno (2)	2	2	1.0 ± 0.5	0.0028 ± 0.0035	2	none	Lowland
Yasuní (8)	5	1	0	0	0	none	Lowland
La Selva (10)	3	1	0	0	0	none	Lowland
Auca 14 (7)	3	2	0.7 ± 0.3	0.1051 ± 0.0790	102	none	Lowland and Southeastern
Lowland	2	1	0	0	0	none	
Southeastern	1	1	1.0 ± 0	0	0	none	
Kapawi (13)	5	1	0	0	0	none	Southeastern

Cyt *b *population diversity values: Locality (and corresponding number from Figure 1), number of individuals sequenced (N), number of haplotypes (*n*), haplotype diversity (*h*), nucleotide diversity (π), polymorphic sites (*s*), cyt *b *haplotype sharing, and clade (determined from phylogenetic analyses). Where more than one clade is found at a particular locality, analyses are shown combined and separated by clade.

The ratio of nonsynonymous to synonymous mutations between all pairs of clades as calculated with a McDonald-Kreitman test does not suggest that selection is responsible for the cyt *b *divergence among lineages (Upland versus Lowland, *P *= 1.0; Upland versus Kapawi, *P *= 1.0; Kapawi versus Lowland, *P *= 0.3). Overall non-significant value of Tajima's *D *(0.255; *P *> 0.1) and within clades (Upland Clade *D *= -1.707, *P *> 0.05, Lowland Clade *D *= -0.834, *P *> 0.1, insufficient sample size to test Southeastern Clade) also implies neutrality (Table 2).

**Table 2:** Historical population expansion by clade

	Upland	Lowland	Southeastern
Mean (obs.)	11.796	5.858	10.000
τ	11.250 (5.356 – 22.945)	2.180 (0.534 – 4.627)	3.000 (0.430 – 3.160)
θ_0_	4.945	0.002	0.219
θ_1_	4.962	4.003	0.290
SSD	0.047 *P *= 0.458	0.044 *P *= 0.056	0.155 *P *= 0.030
Raggedness Index	0.045 *P *= 0.640	0.142 *P *= 0.107	0.667 *P *= 0.620
*t *(MYA)	0.793 (0.378 – 1.618)	0.154 (0.038 – 0.326)	------
Fu's *F*	2.686 *P *= 0.880	-1.547 *P *= 0.324	8.007 *P *= 0.99
Tajima's *D*	-1.707 *P *> 0.05	-0.834 *P *> 0.01	------

Mismatch analysis parameters by clade: mean number of observed differences; expansion parameter τ (with upper and lower bounds at α = 0.05); θ is the substitution rate before (0) and after (1) the expansion; SSD tests the validity of a stepwise expansion model based on the sum of squares deviations between the observed and expected mismatch, with probability values (*P*). Non-significant mismatch values suggest population expansion. Raggedness Index is calculated similarly, and with probability values (*P*). Non-significant raggedness values suggest population expansion. Time since lineage expansion (*t*) is calculated from τ = 2μt, where μ = 1.0%/MYR for 709 bp. Under a model of sudden population expansion, Fu's *F *and Tajima's *D *are expected to be significantly negative. Some parameters are not estimated for the Southeastern Clade because of small sample sizes.

A survey of five tetranucleotide microsatellite loci across individuals of the Upland and Lowland clades in the Napo River area (localities 3 – 11), including a locale where both mitochondrial clades are present, shows that microsatellite genotypes at all loci are very different between Upland and Lowland clades. This implies complete reproductive isolation. The microsatellite library was developed based on Lowland Clade frogs. Consequently, in individuals from the Upland Clade the microsatellite loci are either non-functional (not amplifying after repeated attempts) or allele sizes are non-overlapping or have very different range of sizes, with the Upland Clade always having the larger mean allele size (Table 3). Sequences of large alleles from a subset of samples from three of the five loci (Eloc-Batman&Robin, Eloc-Laurel&Hardy, and Eloc-Thelma&Louise) demonstrate that very large allele sizes in Upland Clade individuals are due to an increase in the number of microsatellite repeats (unpubl. data).

**Table 3:** Microsatellite allele sizes

Locus	Lowland	Upland	Comment
Eloc-Laurel&Hardy	166–262	---	Generally non-amplifying in Upland and twice the repeat length vs. Lowland
Eloc-Bert&Ernie	106–194	202–250	Non-overlapping allele sizes
Eloc-Thelma&Louise	151–247	243–443	Non-overlapping allele sizes except in two individuals
Eloc-Batman&Robin	208–282	300–404	Non-overlapping allele sizes (excluding four Lowland individuals with possible large alleles sizes, 376–464)
Eloc-Beauty&Beast	145–221	185–241	Unequal allele size ranges

Microsatellite profiles (in total fragment size) by locus examined in *E. ockendeni *from the Napo River area (localities 3 – 11). Microsatellite allele sizes (total fragment bp) were larger in the Upland than Lowland Clade. Using *E. ockendeni *primers [105], one locus exclusively amplified in the Lowland Clade, suggesting it is non-functional in the Upland Clade.

### Estimating divergence and expansion

We used three different methods to estimate divergence: the net divergence method to estimate species divergence time; a Bayesian MCMC method to estimate lineage divergence (time to most recent common ancestor, TMRCA); and, a coalescent MCMC approach to estimate migration and TMRCA. Of these, the net divergence method and the Bayesian MCMC method (as implemented in the program BEAST) gave similar temporal results. Divergence estimates from coalescent analyses (as implemented in the program MDIV) were typically less than half the age of the other two estimates.

By the net divergence method, time of divergence between the Upland and Lowland lineages using our slowest estimated rate of evolution (0.6%/MYR) was 20.02 ± 2.87 mya (early Miocene) while the faster rate (1.0%/MYR) estimates the same split at 12.01 ± 1.72 mya (mid-Miocene) (Table 4). Southeastern Clade split from Upland and Lowland clades approximately 25 ± 3 mya (late Oligocene) at the slower substitution rate and 15 ± 2 mya (mid-Miocene) by the faster rate.

**Table 4:** Time of divergence among *E. ockendeni *clades based on net divergence

Clade divergence	MYA (± s.e.) at 0.6%	MYA (± s.e.) at 1.0%
Upland and Lowland	20.02 (17.15 – 22.88)	12.01 (10.29 – 13.73)
Upland and Southeastern	25.75 (22.70 – 28.80)	15.45 (13.62 – 17.28)
Lowland and Southeastern	25.43 (22.27 – 28.60)	15.26 (13.36 – 17.16)

Time of divergence estimates among monophyletic clades in millions of years (mya) calculated from net divergence among clades (upper and lower standard error bounds) assuming 0.6% and 1.0%/MYR substitution rates.

From BEAST, assuming a constant molecular clock and rates of 0.6 and 1.0% substitutions per million years, we estimated the TMRCA of the entire ingroup to be 24.39 mya (late Oligocene) and 14.61 mya (mid-Miocene), respectively. For the TMRCA of the Upland and Lowland clades, the constant clock TMRCA estimates 15.19 mya (mid-Miocene) or 9.11 mya (late Miocene), respectively. The uncorrelated, relaxed clock estimates were not substantially different: assuming mean substitution rates of 0.6 and 1.0%, the TMRCA estimates for the entire ingroup were 27.01 and 15.10 mya, respectively, while those for the Lowland/Upland clade were 15.23 and 9.03 mya, respectively (Table 5).

**Table 5:** Time of divergence among *E. ockendeni *clades based on Bayesian coalescent estimation

		at 0.6%/MYR	at 1.0%/MYR
	clock	TMRCA (mya)	TMRCA (mya)
Ingroup	relaxed	27.01 (15.46–39.01)	15.1 (9.65–20.96)
	constant	24.39 (18.72–30.37)	14.61 (11.39–18.37)
Upland and Lowland	relaxed	15.23 (9.39–21.80)	9.03 (5.79–12.32)
	constant	15.19 (11.71–19.05)	9.11 (7.03–11.39)
Southeastern Clade	relaxed	5.08 (1.79–8.62)	2.70 (1.19–4.81)
	constant	4.53 (2.78–6.30)	2.72 (1.65–3.75)
Upland Clade	relaxed	5.12 (2.44–8.45)	2.88 (1.52–4.49)
	constant	6.25 (4.29–8.26)	2.70 (1.80–3.61)
Lowland Clade	relaxed	6.59 (3.27–10.48)	3.82 (2.01–5.76)
	constant	4.50 (3.09–6.11)	3.75 (2.59–4.94)

Time of divergence estimates among monophyletic clades in millions of years (mya) at 0.6% and 1.0%/MYR substitution rates calculated from Bayesian coalescent phylogenetic estimation of time to most recent common ancestor (with 95% highest posterior density), modelled assuming a relaxed or constant molecular clock (as implemented in BEAST).

Coalescent calculations (implemented in the program MDIV) of the Upland and Lowland clade divergence resulted in an average θ of 6.23 ± 0.13 and an average *T *of 8.78 ± 0.31 (Table 6). Estimates of TMRCA are 7.74 mya assuming a substitution rate of 0.6%/MYR and 4.64 mya assuming the faster substitution rate of 1.0%/MYR. In models of Southeastern Clade versus Upland Clade divergence, θ averaged 8.43 ± 0.27 and *T *7.73 ± 0.52, suggesting a TMRCA of 10.55 mya under the slower substitution rate and 6.33 mya, with the faster rate. Models of Southeastern versus Lowland clades identified an average θ of 9.45 ± 0.38 and an average *T *of 8.98 ± 0.69, or 9.99 mya or 7.77 mya TMRCA, depending on substitution rate. For all clade comparisons *M *modelled best as 0 suggesting there is no gene flow among clades. The estimated time since population divergence among all clades is less than the TMRCA. MDIV estimates of TMRCA and species divergence are non-overlapping with BEAST and the net divergence method.

**Table 6:** Estimates of time since population divergence (*T*_pop_) and time to most recent common ancestor (*T*_MRCA_), inferred using a coalescent MCMC approach to estimate migration and divergence

Coalescence Divergence Time (MDIV)			
		at 0.6%/MYR	at 1.0%/MYR
Clade comparison		mya	mya
Upland vs. Lowland	*T*_pop_	6.45 ± 0.11	3.87 ± 0.07
	*T*_MRCA_	7.74 ± 0.17	4.64 ± 0.10
Upland vs. Southeastern	*T*_pop_	7.68 ± 0.60	4.61 ± 0.36
	*T*_MRCA_	10.55 ± 0.20	6.33 ± 0.12
Lowland vs. Southeastern	*T*_pop_	9.99 ± 0.64	5.99 ± 0.38
	*T*_MRCA_	12.96 ± 0.66	7.77 ± 0.40

Time estimates are based on substitution rates of 0.6%/MYR and 1.0%/MYR.

### Estimating population expansion

We used four different methods to try and identify and estimate the timing of population expansion. Based on mismatch analyses, the sudden expansion model of population growth cannot be rejected for the Upland or the Lowland clades, although the SSD probability was marginal for the Lowland Clade (*P *= 0.056) (Table 2). Using a mutation rate of 1.0%/MYR and the peak of the mismatch distribution, τ, to estimate the time of population expansion suggests an Upland Clade expansion began in the latter half of the Pleistocene (793 000 YBP), a Lowland Clade expansion (if it occurred) somewhat later (154 000 YBP). The model of sudden population expansion is rejected for the Southeastern Clade. Raggedness indices suggest population expansion (curves are not significantly different than smooth) in all three clades. Fu's *F *and Tajima's *D *are not different than would be found under a stable population size. Therefore, we have conflicting evidence from different methods but some suggestion of population expansion particularly in the Upland Clade.

## Discussion

### Phylogenetic and phylogeographic relationships

Divergence among the three strongly supported clades in Ecuadorean *E. ockendeni *is deep and well supported (Figure 2, Additional file 2). In contrast, there is relatively low divergence within the three clades. The Southeastern Clade is basal relative to the Upland and Lowland clades. The timing of the divergences for all three lineages suggests causal events that vastly predate the Pleistocene and the latter phases of orogenesis in the northern Andes. We discuss these below.

Five independent nuclear markers corroborate that the Upland and Lowland clades are evolutionarily distinct. The microsatellite loci were either non-amplifying or composed of twice as many repeats in the Upland as Lowland Clade, even in frogs from the same geographic locality (Table 3). Such differentiation at microsatellite loci between clades even when they are sympatric and the potential for interbreeding exists provides strong indirect support that these are reproductively isolated. Unfortunately, we still know little of the mate recognition system for this complex of species.

The major *E. ockendeni *clades are geographically restricted and found in the east Andean uplands (approx. 400 to 1000 masl; Upland Clade), the central and northern lowlands (220 to 500 masl; Lowland Clade), and the central and southern lowlands (260 to 240 masl; Southeastern Clade). Our sampling is limited to Ecuador and therefore we cannot infer whether the clades are more broadly distributed. The headwaters of the Napo River appears to be part of a lineage contact zone: at EBJS (locality 5) both the Upland and Lowland Clade can be found; slightly upriver at Serena (3) and Cando (4), the Lowland Clade is found on the south side of the Napo River while the Upland Clade is found immediately on the opposite side of the river. Further east at Auca 14 Road (locality 7), again two lineages can be found sympatrically (Upland and Southeastern clades). At all other geographic localities sampled we found only one of the three clades. *Physalaemus petersi *was also found to have an upper Napo and lower Napo genetic division and a basal southeastern lineage [7]. Further, an upper and lower Napo basin phylogeographic break has been suggested in *Bolitoglossa *salamanders [31]. Therefore, evidence from other amphibians may indicate this to be a cryptic geographic break, as has been found in other upper Amazon localities [4].

In *E. ockendeni*, the lack of haplotypic diversity at many localities and general lack of haplotype sharing among localities suggests fine-scale restricted gene flow (Table 1), as might be expected from a small terrestrial amphibian [27]. Population structure at finer geographic scales in the upper Amazon has also suggested restricted gene flow in this species [32].

We have included *E. ockendeni *from Cuyabeno (locality 2) in northeastern Ecuador in the Lowland Clade; however, they do display a notable genetic divergence from the rest of the Lowland Clade (> 6% corrected p-distance; Additional file 2) in a group that is otherwise characterized by low within-clade diversity. Further sampling from the surrounding geographic area (e.g., northern Peru and southern Colombia) and more molecular markers will either strengthen our inclusion of Cuyabeno *E. ockendeni *in the Lowland Clade or differentiate it as a separate clade.

### Time of divergence

Our divergence estimates suggest that diversification among the "species" *E. ockendeni *is ancient and predates the Andes at their current height in Ecuador. Divergence between the Southeastern and Upland/Lowland lineages occurred in the late Oligocene or early Miocene and between the Upland and Lowland Clade in the early, mid-, or late Miocene, depending on substitution rate.

Of the three estimates, we consider the Bayesian phylogenetic-coalescent method (Table 5) to be the most accurate because that method accommodates the greatest number of parameters, incorporates molecular evolutionary complexities such as rate heterogeneity, allows for differences in rates among lineages, and allows for tests of the molecular clock [33]. Interestingly, and in support of a long-standing method, the much less complicated net divergence method (Table 4) has yielded very similar species divergence time estimates to the Bayesian method TMRCA. We suggest that the MDIV coalescent method (Table 6) is underestimating time of divergence in this case, perhaps because there is not enough historical information to suit a population coalescent method when species are reciprocally monophyletic and deeply diverged, or because MDIV does not accommodate rate variation among sites.

Some studies have found the rate of molecular evolution in the tropics to be equivalent to temperate areas [34,35] while others have suggested a faster rate of molecular evolution in the tropics [36-38]. Obviously, the timing of the divergence among *E. ockendeni *clades will become younger given the same genetic distance if true substitution rate is faster than we have estimated.

### Historical population change

Across three different tests, there is weak and somewhat conflicting suggestion of recent population expansion under models of neutral evolution (Table 2). For the Upland Clade, we cannot reject sudden expansion from mismatch distributions or raggedness though Fu's *F *and Tajima's *D *do not suggest population expansion. For the Lowland Clade, we can probably reject population expansion since mismatch and raggedness significance values are only barely non-significant (*P *= 0.056 for mismatch, *P *= 0.107 for raggedness) and *F *and *D *do not suggest expansion. In the Southeastern Clade, sample sizes are very small (two localities, two haplotypes), but mismatch and *F *do not suggest population expansion while raggedness index does fit the expectation of expansion.

Our results give some support for an Upland Clade expansion in *E. ockendeni *approximately 800 000 years ago. It is difficult to determine historical population expansion; inconclusive findings from multiple methods are not unexpected because rate heterogeneity, the scale of historical population expansion if there were any, sample size, and number of polymorphic sites are all influential factors [39]. If there has been expansion in the Lowland Clade, this may have occurred 150 000 years ago. In upper Amazonian clades of the terrestrial frog *P. petersi*, inconclusive results about a population expansion have also been found and, like our results here, some support for an Upper Napo clade expansion [7].

### Geological history and its influence on phylogeographic patterns

The upper Amazon of Ecuador has a history of variations in climate and dramatic geology. The northern Andes are the youngest in the Andean chain: orogenesis occurred in the Neogene (Miocene through Holocene) [40], with the principle period of upheaval only in the past five million years [40-42].

Throughout the Miocene there was a radical reorganization of drainage patterns in northern South America. In the early Miocene the fluvial system drained to the northwest but by the late Miocene the effects of the rising Eastern Cordillera shifted drainage eastwards, ultimately allowing the Amazon River to reach the Atlantic [41]. Episodic marine incursions as a result of eustatic sea level changes occurred throughout the Miocene [24,41]. In the Pliocene, Pleistocene, and Holocene, fluvial deposits of many sorts and very large alluvial fans (megafans) were laid throughout the upper Amazon in the wake of Andean tectonism, volcanism, and later glaciation ([41,43-45] and references therein). These overlays and heterogeneity often leave conflicting evidence and cause geological events east of the Andes to be poorly dated [43,46].

Climate also has varied over time, with great fluctuations throughout the Tertiary and Quaternary [47]. Alternating wet and dry cycles in the Pleistocene and Holocene may have caused forest contractions and expansions in some areas, though there is disagreement on the extent of this effect in the upper Amazon [21,47-49]. These forest contracts are the source of the refuge hypothesis of Amazonian diversification [20].

Clearly there are multiple possible ancient historical geological and climatic influences causing isolation and subsequent expansion in *E. ockendeni*, ultimately resulting in the speciation we see here. We can infer that diversification between clades of *E. ockendeni *predates the altitude and shape of the Andes as we know them. Instead, diversification is much older, perhaps precipitated by dramatic changes in the Miocene. For example, mountain-building caused numerous thrust faults to develop and the eastern Subandean Zone fault ([45] and references therein) approximately matches the geographic location of our Upland/Lowland break between clades of *E. ockendeni *and may be historically relevant. Geological change in the Miocene has recently been suggested as influential in South American *Eleutherodactylus *frog diversification in general [50]. Importantly, there is no obvious contemporary barrier between the extant clades of *E. ockendeni*, such as a major river as would be suggested by the river barrier hypothesis [13-16]). The current lack of elevation difference in the distribution of two of the three clades suggests that elevational gradient differences are not driving the divergence (e.g., under an environmental gradient hypothesis [51,52]). Contemporary complex topography (as suggested by, for example [27,53]) does not seem to be relevant to patterns of deep divergence among clades, since divergence far predates existing topography, clades are not isolated by major topographic features, and the distribution of the Upland Clade extends through the headwaters of at least three major river valleys (Agua Rico, Napo, and Pastaza). Further, the genetic diversity in *E. ockendeni *dates from the Miocene and therefore cannot be attributed to climatic and associated vegetation changes of the past two million years, as has been suggested by proponents of the refuge theory [11,20,54]. However, recent population expansion in the Upland Clade may coincide with late Pleistocene climate change.

### Cryptic species richness in *E. ockendeni*

Our findings show that the leaflitter frog species *E. ockendeni *is three distinct species with apparently extreme morphological conservativism. Furthermore, this area is only a portion of the species' range, which extends from southern Colombia to southern Peru [52] and Bolivia [55], so the actual species diversity and richness within "*E. ockendeni*" is likely much greater than that demonstrated here. When the specimens were collected and catalogued for museum deposition, there was no apparent morphological difference among specimens and there has been no published indication that there would be multiple cryptic species within this species, except for a mention that *E. ockendeni *from Cuisime (southern Ecuador) are smaller in snout-vent length than those collected in other areas of Ecuador and Peru [56]. It is clear that the divergence among each of the three clades is biologically real and not an artefact of stochastic variance in mtDNA masking recent gene flow [57] because our coalescent methods model best with no gene flow among lineages (in MDIV *M *= 0) and five nuclear microsatellite loci are significantly different between the Upland and Lowland clades. Given this new molecular information, a detailed morphological revision of Ecuadorean *E. ockendeni *is underway (Elmer and Cannatella, unpubl.).

Incidences of sympatric, parapatric, and allopatric cryptic species have been recently discovered in southeast Asian frogs ([58] and references within). Also, although there are exceedingly few intraspecific molecular phylogenies of neotropical amphibians, those that do exist tend to encounter new species and/or previously unanticipated species diversity ([9,59,60] and references within). These findings together suggest that widespread species of amphibians in the tropics have an evolutionarily history that is much more complicated than suggested by morphologies. Consequently, attempts at biological conservation according to current estimates of the number of morphological "species" will drastically underestimate the actual biodiversity in this already species-rich region.

## Conclusion

We found deep phylogenetic divisions among clades in this common leaflitter frog, *E. ockendeni*, suggestive of distinct species. Based on microsatellite genotype profiles for distinct mitochondrial clades and modelling of historical gene flow, we suggest that there is complete reproductive isolation among these clades, even when they are sympatric. These cryptic species have an ancient divergence estimated to have originated in the Miocene. Diversification among these clades coincides approximately with periods of dramatic northern Andean orogenesis and predates the Andes at their current height. Though multiple environmental occurrences surely have been historically influential, Pleistocene climate change refuges as drivers of allopatric speciation are not relevant to extant specific diversification *E. ockendeni*. Our research strongly suggests that current estimates for the renowned species richness in the upper Amazon of frogs in general and *Eleutherodactylus *in particular may be a substantial underestimate of the actual phyletic diversity present.

## Methods

### Field and Laboratory Methodology

*Eleutherodactylus ockendeni *is a small terrestrial leaflitter frog that is relatively abundant at many upper Amazon localities [32,61,62]. Its range extends throughout the upper Amazon of Colombia, Peru, Ecuador, Brazil [63], and Bolivia [55]. We collected from 12 localities across eastern Ecuador (Figure 1) in 2001 and 2003. Inter-locality distances ranged from 1 to 300 km apart (straight-line distance) and included all major river basins in Ecuador. Individuals were euthanized using MS-222 and tissue samples for genetic analyses removed and stored in pure ethanol. Specimens were fixed with 10% formalin and stored in 70% ethanol. Vouchers are deposited at the Museo de Zoología, Pontificia Universidad Católica del Ecuador (QCAZ) (Additional file 1). Samples from the Parque Nacional Yasuní locality were taken from the existing QCAZ collection.

Genomic DNA was extracted using either standard phenol-chloroform methods [64] or a Qiagen DNEasy kit according to the manufacturer's protocol. Two mtDNA fragments were amplified: 16S rRNA with primers 16Sar-L and 16Sbr-H (ca. 560 bp; numbers 88 and 96 in [65]) and cyt *b *with primers MVZ15L and MVZ16H (ca. 790 bp; numbers 141 and 165 in [65]). Volumes for each PCR reaction were 50 μL and contained: 0.3 μM of forward and reverse primer, PCR enhancing buffer (2.5 mM MgCl2, 10 mM Tris pH 8.4, 50 mM KCl, 0.02 mg BSA, 0.01% gelatin; adapted from [66]), 0.3 mM dNTP, 0.625 units taq DNA polymerase (Fermentas Life Sciences), and approximately 1 to 3 ng stock DNA. All reaction sets included a negative control. Cycling parameters for 16S were: 94°C for 2 min, 35 cycles of denaturing 94°C for 30 sec, annealing 60°C for 20 sec and extending 72°C for 20 sec, a final extension at 72°C for 5 min followed by extended cooling at 10 or 4°C. Cyt *b *parameters were: initial denaturing at 92°C for 3 min, 38 cycles denaturing 92°C for 1 min, annealing 51°C for 1 min and extension 72°C for 1 min, a final extension of 72°C for 5 min followed by extended cooling at 4 or 10°C. PCR product was cleaned for sequencing using a Qiaquick Gel Extraction kit according to the manufacturers' instructions for agarose electrophoresis-separated fragments or Pall AcroPrep 96 Filter Plates for PCR products that were not electrophoresed. Samples were capillary sequenced using the BigDye Terminator version 3.1 Cycle Sequencing (Applied Biosystems) chemistry on an Applied Biosystems 3100 or 3730XL Gene Analyzer.

Forty-seven individuals were sequenced for 16S: 23 in both directions and the remainder only in the forward direction. One hundred and seven individuals were sequenced for cyt *b*: 41 in both directions and the rest only in the forward direction (GenBank accession numbers: 16S EU130581 – EU130626, EU130579, EU130580; cyt *b *EF581013–EF581063, EU130577, EU130578, EU130627–EU130680). Detailed comparison by eye of forward and reverse sequences in the overlapping regions showed no discordance in the DNA sequence used in subsequent analyses. Thirty-two individuals plus outgroup taxa had sequences for both 16S and cyt *b *(hereafter, 16S-cytb). In the 16S-cytb fragment, concatenated sequences were trimmed to equal length (1248 bp), except for five sequences that remained shorter (≥ 1197 bp). In the cyt *b *fragment, all haplotypes were the same length (709 bp) except three (≥ 647 bp). Terminal gaps were coded as missing data.

### Phylogenetic Analyses

Sequences were assembled in MACCLADE version 4.07 [67] and aligned in CLUSTAL X version 1.81 [68] using default settings. A hyper-variable loop portion of the 16S alignment could not be aligned with confidence, so nine internal positions were excluded. Alignment of cyt *b *was not problematic and included no internal gaps. The best-fit models of nucleotide substitution of cytochrome *b*, each codon position separately, and 16S were estimated using MODELTEST version 3.7 [69] in PAUP* [70].

Because "intraspecific" diversity in these frogs is so high, we analyzed these data using maximum parsimony (MP) and Bayesian phylogenetic tree methods (rather than network methods [71]). Before MP analysis, we performed a partition homogeneity test [72] with 100 replicates and 10 random addition replicates per replicate in PAUP* [70] to assess whether 16S and cyt *b *have congruent phylogenetic signal. The 16S-cytb fragment MP analyses were run in PAUP* with outgroups *Eleutherodactylus acuminatus *and *Eleutherodactylus quaquaversus *(*unistrigatus *group [73]), using a heuristic search strategy with TBR branch-swapping and 1000 random addition replicates. The shortest tree was saved from each replicate. The topology of the 11 most parsimonious trees was tested with 1000 heuristic search bootstrap pseudoreplicates, with 10 random-addition replicates each, and merged into a strict 50% consensus tree. MP analyses were repeated with the full cyt *b *and 16S data sets separately. For our Bayesian phylogenetic analyses, we used a two partition (16S and cyt *b*) analysis in MRBAYES version 3.1.2 [74] with likelihood parameters nst = 6, rates = gamma. Two independent MCMC chains were run for 4.8 million generations with reduced temperature difference among chains (t = 0.09) and trees sampled every 100 generations. After assessing for apparent convergence [75], 25 000 trees were discarded as burn-in and a 50% majority-rule consensus tree was built.

### Time of divergence estimates

We used cyt *b *to estimate divergence times because we had more data for this fragment (individuals and haplotypes), there are more published estimates of rate of evolution available than 16S, and cyt *b *is less subject to inter-study variation because of alignment-specific exclusion of hyper-variable regions typical of rDNA alignments. Divergence time among lineages or species was estimated in three ways. First, net among clade divergence ([30] p. 276) ± standard error corrected with a TN model [76] of nucleotide substitution was calculated in MEGA3 [77]. This method subtracts the intraspecific diversity from overall divergence between two species and is unbiased when lineages are reciprocally monophyletic and ancestral population sizes is equal to the average of the two descendent species [reviewed in [78]]. Species divergence time was calculated from the net divergence estimate (net divergence × substitutions/site/MYR). The TN model is a more general case of the HKY model [79], which is implemented in MDIV (below) and was selected as an appropriate model of sequence evolution in MODELTEST.

Second, we estimated lineage divergence time, population divergence, and migration rate between major pairwise clades in MDIV [80] on CBSU Web Computing Resources. Initial runs were tested under a finite sites (HKY) model of evolution and default priors (*M *= 10, *T *= 5) to approximate the posterior distribution of scaled migration rate (*M*) and time since divergence (*T*), while allowing MDIV to estimate θ. Once appropriate parameter values to bound a "well-behaved" posterior distribution [80] were identified (*M *= 0, *T *= 30), we ran the MCMC for two million generations with 500 000 generations discarded as burn-in. Convergence was determined by evaluating the consistency of model values for each of the three parameters across five runs, which were then averaged to calculate mean θ and *T *values ± standard deviation. Time of divergence was calculated as (following [81]): *t*_div _= (*T*θ/2L)/(1/μ) where *T *(or TMRCA) and θ were estimated by the height of the posterior distribution, L is the sequence length analyzed (709 bp of cyt *b*), and μ is the mutation rate (here, substitution rate). Substitution rates may be less than mutation rates because neutral mutation rates include population polymorphism that will not eventually be fixed in phylogenetic lineages (reviewed in [82,83] but see [84]). Given the high levels of divergence among lineages here, we consider substitution rate more realistic than mutation rate.

As a third approach to calculating the timing of diversification, we estimated time to most recent common ancestor (TMRCA) for various clades using a Bayesian approach with the program BEAST version 1.4.1 [85]. All analyses were performed using an HKY model of nucleotide substitution with gamma distributed rate variation among sites and six rate categories. We ran four separate sets of analyses, first assuming a constant population size and a constant global molecular clock (i.e. no rate differences among lineages) of either 0.6 or 1.0%/MYR, and second using an uncorrelated, relaxed clock again assuming constant population size and mean clock rates of either 0.6 or 1.0%/MYR. Results from two independent runs (10,000,000 generations with the first 1,000,000 discarded as burn-in and parameter values sampled every 1000 generations) for each combination of settings were combined and the effective sample size for parameter estimates and convergence checked using the program Tracer version 1.3 [86].

The substitution rate of *E. ockendeni *cyt *b *is unknown, so for all three methods we used the same two estimates of 0.6 and 1.0 substitutions/site/100 MYR. We inferred our substitution rate from two lines of evidence. First, the mtDNA gene ND2 has been found to be consistent across diverse poikilothermic vertebrate lineages (such as fish, frogs, lizards, and salamanders [87]) and to have a substitution rate similar to cyt *b *[88]. A mean ND2 substitution rate of 0.957%/MYR has been suggested in Costa Rican *Eleutherodactylus *inferred from Eurasian toads [89]. Second, multiple calibrated salamander studies have found cyt *b *substitution rates in the range of 0.6 to 0.8%/MYR [90-92]. Frogs and salamanders share many characteristics that might be important in determining molecular rates of evolution (e.g., generation time, body size, ectothermy [92-94] and constancy among taxa has been shown in other mtDNA genes [87], so salamander substitution rate can be considered a reasonable approximation for frog substitution rate.

### Population genetics analyses – mtDNA

We used a McDonald-Kreitman approach [95] to test for selection among clades and calculated Tajima's *D *[96] to test for neutrality in DNASP version 4.10.4 [97]. Tajima's *D *is also used for estimating population expansion.

Cyt *b *haplotypic (gene) diversity, number of polymorphic sites, and nucleotide diversity [30] per locality were calculated in ARLEQUIN v. 2.000 [98]. Inter-haplotype TN corrected p-distances were calculated in MEGA3 [77]. Cyt *b *mismatch distributions and raggedness index (*r*) [99] were calculated by clade in ARLEQUIN under a model of sudden expansion using the parametric bootstrap approach (α = 0.05; 1000 bootstraps) [100]. An empirical mismatch distribution that does not deviate from a unimodal distribution of pairwise differences among haplotypes and has a smooth distribution [99] suggests recent population expansion [101,102]. The beginning of the population expansion can be estimated from τ, the crest of the mismatch distribution: τ = 2μt [102], where t is the generation time (unknown, but estimated as 1 year [89]) and μ is the upper estimate divergence rate (1.0%/MYR × number of bp). As an alternative test of population expansion, in ARLEQUIN we calculated Fu's *F *[103] and tested its significant with 1000 bootstrap replications. Significantly negative values of Fu's *F *suggest population expansion [104].

### Population genetics analyses – microsatellites

Five microsatellite loci (Eloc-Bert&Ernie, Eloc-Beauty&Beast; Eloc-Thelma&Louise; Eloc-Laurel&Hardy and Eloc-Batman&Robin) [105] were amplified for all individuals in the Napo River area (localities 3 – 11 in Figure 1) for which we also have mtDNA sequences (n = 21 from Upland Clade; n = 76 from Lowland Clade). Samples were amplified and genotyped using published conditions [105]. Amplification and scoring of a subset of samples was repeated to confirm genotypes. Four samples that resulted in large microsatellites in the loci Eloc-Thelma&Louise and Eloc-Batman&Robin were sequenced on an ABI 310 capillary sequencer using the microsatellite primers to determine exact composition of the microsatellite. A homozygous individual for locus Eloc-Laurel&Hardy was amplified by PCR and cloned using pGEM-T Vector System II kit (Promega) and the inserts sequenced in an ABI 3100 sequencer using M13 primers.

## Authors' contributions

KRE designed the study, collected samples, carried out the molecular studies, performed statistical analyses, and drafted the manuscript. JAD assisted with microsatellite primer design, genotyping, and microsatellite analyses, sequenced microsatellites, and interpreted results. SCL participated in the design of the study, performed statistical analyses, and helped draft the manuscript. All authors read and approved the final manuscript.

## Supplementary Material

Additional file 1 Specimen information. Museum catalogue numbers (QCAZ) for *E. ockendeni *and two outgroup species, GenBank accession numbers for cyt b and/or 16S fragment, and locality of origin for each individual used in the study. Click here for file

Additional file 2 TN-corrected p-distance among cyt b haplotypes. TN-corrected p-distances among cyt b haplotypes of *E. ockendeni*, grouped by clade. Click here for file
