# Supplementary material for: Cryptic diversity and deep divergence in an upper Amazonian leaflitter frog, Eleutherodactylus ockendeni
Source: BMC Evol Biol. 2007 Dec 21;7:247. doi: 10.1186/1471-2148-7-247 (PMC2254618; doi:10.1186/1471-2148-7-247)
Supplement: Additional file 1 — Specimen information. Museum catalogue numbers (QCAZ) for E. ockendeni and two outgroup species, GenBank accession numbers for cyt b and/or 16S fragment, and locality of origin for each individual used in the study. [file 1471-2148-7-247-S1.pdf]

Additional file 1.

QCAZ	Cyt *b*	16S	Locality
14891		EU130586	Yasuní
14893		EU130589	Yasuní
14895	EU130632	EU130587	Yasuní
14896	EU130631		Yasuní
14897	EU130630		Yasuní
14898	EU130633	EU130588	Yasuní
18023		EU130581	EBJS
18027	EF581014		EBJS
18030	EF581015		EBJS
18033	EF581016		EBJS
18039	EF581017		EBJS
18041	EF581018		EBJS
18045	EF581019		EBJS
18053	EF581020		EBJS
18069	EF581021	EU130582	EBJS
18071	EF581022		EBJS
18077	EF581023		EBJS
18085	EF581024		EBJS
18089	EF581025		EBJS
18092	EF581026		EBJS
18096	EF581027		EBJS
18098	EF581028		EBJS
18110	EF581029		EBJS
18112	EF581030		EBJS
18117	EF581031		EBJS
18126	EF581032		EBJS
18128	EF581033		EBJS
18132	EF581034	EU130583	EBJS
18137	EF581035		EBJS
18139	EF581036		EBJS
18148	EU130627		EBJS
18153	EF581037		EBJS
18156	EU130628	EU130584	Puca Chicta
18157	EU130629	EU130585	Puca Chicta
18165	EF581013		EBJS
19208	EU130678		Yasuní
25165	EU130673	EU130622	Hola Vida
25273	EU130679	EU130626	Auca 14
25277	EU130680	EU130594	Auca 14
25287	EF581048		EBJS
25288	EF581049		EBJS
25290	EF581051		EBJS
25291	EF581050		EBJS
25295	EF581052	EU130595	EBJS
25306	EU130650	EU130606	Cando
25307	EU130651		Cando
25308	EU130652		Cando

25309	EU130653	EU130607	Cando
25310	EU130654		Cando
25313		EU130608	Cando
25324	EU130647	EU130605	Cando
25325	EU130648		Cando
25326	EU130649		Cando
25327	EU130655	EU130609	Llanganates
25328	EU130656	EU130610	Llanganates
25337		EU130611	Santa Clara
25343	EU130657	EU130612	Santa Clara
25344	EU130658		Santa Clara
25345	EU130659		Santa Clara
25348	EU130660		Santa Clara
25349	EU130661	EU130613	Santa Clara
25372		EU130614	Santa Clara
25427	EU130662	EU130615	La Selva
25428	EU130663	EU130616	La Selva
25429	EU130664		La Selva
25439	EU130665	EU130617	EBJS
25445	EU130666		EBJS
25447	EU130667		EBJS
25457	EU130668	EU130618	Kapawi
25458	EU130669		Kapawi
25459	EU130670		Kapawi
25462		EU130619	Kapawi
25492		EU130620	Kapawi
25526	EU130671		Kapawi
25527	EU130672	EU130621	Kapawi
25552	EF581039		EBJS
25554	EF581040		EBJS
25555	EF581038		EBJS
25558	EF581041		EBJS
25561	EF581042		EBJS
25564	EF581043		EBJS
25573	EF581044		EBJS
25575	EF581045		EBJS
25576	EU130634	EU130590	Cuyabeno
25577	EU130635	EU130591	Cuyabeno
25579	EF581046	EU130592	EBJS
25580	EF581047		EBJS
25599	EU130636	EU130593	Auca 14
25601	EU130674		Hola Vida
25654	EU130675	EU130623	Hola Vida
25655	EU130676		Hola Vida
25658		EU130624	Hola Vida
25660	EU130677		Hola Vida
25700		EU130625	Hola Vida
25751	EF581055		EBJS
25756	EF581056		EBJS
25757	EF581054		EBJS
25759	EF581053	EU130596	EBJS

25764	EF581057		EBJS
25770	EF581058		EBJS
25773	EF581060		EBJS
25774	EF581061		EBJS
25776	EF581059		EBJS
25782	EF581062		EBJS
25783	EF581063		EBJS
25807	EU130637		Chonta Yacu
25808	EU130638	EU130597	Chonta Yacu
25809	EU130639		Chonta Yacu
25810	EU130640	EU130598	Chonta Yacu
25811	EU130641		Chonta Yacu
25831		EU130599	Chonta Yacu
25838		EU130600	Chonta Yacu
25839	EU130642	EU130601	Serena
25842		EU130603	Serena
25845	EU130643		Serena
25846	EU130644		Serena
25847	EU130646	EU130604	Serena
25854	EU130645		Serena
25855		EU130602	Serena
19664	EU130577	EU130579	*E. acuminatus* Yasuní
25613	EU130578	EU130580	*E. quaquaversus* Hola Vida
