# Supplementary material for: Cryptic diversity and deep divergence in an upper Amazonian leaflitter frog, Eleutherodactylus ockendeni
Source: BMC Evol Biol. 2007 Dec 21;7:247. doi: 10.1186/1471-2148-7-247 (PMC2254618; doi:10.1186/1471-2148-7-247)
Supplement: Additional file 2 — TN-corrected p-distance among cyt b haplotypes. TN-corrected p-distances among cyt b haplotypes of E. ockendeni, grouped by clade. [file 1471-2148-7-247-S2.pdf]

Clade			1	2	3	4	5	6	7	8	9	10	11	12	13	14	15	16	17	18	19	20
South-eastern <	1	E. *ockenden*i Kapawi																				
	2	*E. ockendeni* Auca 14	0.0482																			
Lowland	3	*E. ockendeni* EBJS/Puca Chicta	0.1699	0.1835																		
	4	*E. ockendeni* EBJS	0.1722	0.1858	0.0016																	
	5	*E. ockendeni* EBJS	0.1699	0.1835	0.0000	0.0016																
	6	*E. ockendeni* Serena/Puca Chicta	0.1722	0.1858	0.0016	0.0033	0.0016															
	7	*E. ockendeni* EBJS	0.1699	0.1835	0.0000	0.0016	0.0000	0.0016														
	8	*E. ockendeni* EBJS	0.1743	0.1879	0.0033	0.0016	0.0033	0.0049	0.0033													
	9	*E. ockendeni* Yasuní	0.1678	0.1814	0.0016	0.0033	0.0016	0.0033	0.0016	0.0049												
	10	*E. ockendeni* La Selva	0.1719	0.1854	0.0016	0.0033	0.0016	0.0033	0.0016	0.0049	0.0033											
	11	*E. ockendeni* Auca 14	0.1699	0.1835	0.0000	0.0016	0.0000	0.0016	0.0000	0.0033	0.0016	0.0016										
	12	*E. ockendeni* Cuyabeno	0.1635	0.1750	0.0604	0.0622	0.0604	0.0622	0.0604	0.0641	0.0585	0.0621	0.0604									
	13	*E. ockendeni* Cuyabeno	0.1635	0.1750	0.0604	0.0622	0.0604	0.0622	0.0604	0.0641	0.0585	0.0621	0.0604	0.0033								
Upland	14	*E. ockendeni* EBJS	0.1950	0.2031	0.1383	0.1383	0.1383	0.1361	0.1383	0.1403	0.1363	0.1402	0.1383	0.1488	0.1465							
	15	*E. ockendeni* Chonta Yacu	0.1851	0.1986	0.1320	0.1320	0.1320	0.1342	0.1320	0.1340	0.1301	0.1339	0.1320	0.1382	0.1360	0.0495						
	16	*E. ockendeni* Llanganates	0.1995	0.2077	0.1363	0.1363	0.1363	0.1341	0.1363	0.1383	0.1343	0.1381	0.1363	0.1508	0.1485	0.0049	0.0513					
	17	*E. ockendeni* Cando/Llang../Sta Clara	0.1950	0.2031	0.1361	0.1361	0.1361	0.1339	0.1361	0.1381	0.1341	0.1380	0.1361	0.1465	0.1443	0.0016	0.0513	0.0033				
	18	*E. ockendeni* Santa Clara	0.1950	0.2031	0.1361	0.1361	0.1361	0.1339	0.1361	0.1381	0.1341	0.1380	0.1361	0.1465	0.1443	0.0049	0.0550	0.0066	0.0033			
	19	*E. ockendeni* Santa Clara	0.1995	0.2077	0.1403	0.1403	0.1403	0.1381	0.1403	0.1423	0.1383	0.1422	0.1403	0.1468	0.1446	0.0049	0.0550	0.0066	0.0033	0.0033		
	20	*E. ockendeni* Santa Clara	0.1974	0.2056	0.1383	0.1383	0.1383	0.1361	0.1383	0.1403	0.1363	0.1402	0.1383	0.1488	0.1465	0.0033	0.0532	0.0049	0.0016	0.0016	0.0016	
	21	*E. ockendeni* Hola Vida	0.1904	0.2026	0.1339	0.1339	0.1339	0.1318	0.1339	0.1359	0.1319	0.1358	0.1339	0.1404	0.1382	0.0099	0.0513	0.0116	0.0082	0.0116	0.0116	0.0099
